# Chemotherapy-Induced Alopecia in Ovarian Cancer: Incidence, Mechanisms, and Impact Across Treatment Regimens

**DOI:** 10.3390/cancers17030411

**Published:** 2025-01-26

**Authors:** Simonetta I. Gaumond, Gabriela E. Beraja, Isabella Kamholtz, Lina M. Ferrari, Rami H. Mahmoud, Joaquin J. Jimenez

**Affiliations:** 1 Department of Biochemistry and Molecular Biology, University of Miami Miller School of Medicine, Miami, FL 33136, USA; 2 Dr. Phillip Frost Department of Dermatology and Cutaneous Surgery, University of Miami Miller School of Medicine, Miami, FL 33136, USA

**Keywords:** chemotherapy-induced alopecia, chemotherapy, ovarian cancer, alopecia, hair loss, taxanes, platinum compounds

## Abstract

Background/Objectives: Ovarian cancer is the fifth most common cancer among women, with an estimated 19,680 new cases projected in 2024. Adjuvant chemotherapy remains the standard treatment for epithelial ovarian cancers but is frequently associated with adverse events, such as chemotherapy-induced alopecia (CIA). CIA is a particularly distressing side effect that significantly affects the body image, self-esteem, and quality of life of patients. Unfortunately, CIA remains underexplored in patients with ovarian cancer. Methods: This scoping review analyzed PubMed- and EMBASE-indexed studies investigating the incidence, severity, and mechanisms of CIA in ovarian cancer patients. Eighteen studies were included for analysis. Results: Our analysis identified platinum-based compounds, taxanes, and topoisomerase I inhibitors as the agents most strongly correlated with severe alopecia, particularly in combination regimens such as carboplatin–paclitaxel (CP), and cyclophosphamide, adriamycin, and cisplatin (CAP). Among the monotherapies, taxanes, including paclitaxel and docetaxel, posed the highest risk of CIA. Mild-to-moderate alopecia was observed in patients treated with gemcitabine or pegylated liposomal doxorubicin. Alternative factors such as dosing schedules and prior chemotherapy exposure also significantly influence CIA severity. Conclusions: Given the profound psychosocial impact of CIA, optimizing treatment protocols to reduce the severity of alopecia without compromising therapeutic efficacy is crucial. These findings offer insights that may guide future therapeutic strategies for improving patient outcomes and quality of life.

## Simple Summary

Chemotherapy-induced alopecia (CIA) is a common and distressing side effect of cancer treatment, significantly impacting patients’ self-esteem and quality of life. While chemotherapy is a cornerstone of ovarian cancer care, its impact on hair loss has not been comprehensively studied in this patient population. This review examines the frequency and severity of CIA associated with widely used chemotherapies, including taxanes and platinum-based agents, and evaluates factors such as drug combinations and dosing schedules that influence hair loss. By identifying patterns and potential strategies to minimize CIA, this study aims to improve patient quality of life without compromising the effectiveness of cancer treatments. These findings provide valuable insights for clinicians and their patients, fostering informed decision-making and encouraging further advancements in managing the adverse effects of cancer therapies.

## 1. Introduction

Ovarian cancer is the fifth most common cancer among women, with an estimated 19,680 new cases projected for 2024 and a 5-year relative survival rate of 50.9% according to the NIH National Cancer Institute. Ovarian tumors are categorized into three major types: surface epithelial–stromal tumors, sex cord–stromal tumors, and germ cell tumors [1]. Epithelial ovarian cancers account for approximately 95% of ovarian cancer cases [2].

The absence of early specific symptoms and effective screening methods often results in late-stage diagnosis, with most patients presenting with stage III or IV disease, characterized by metastasis beyond the pelvis [3,4]. In contrast, early detection of stage I or II ovarian cancer, followed by surgery and chemotherapy, achieves cure rates of 70–90% [5]. However, late-stage diagnosis reduces the cure rate by approximately 20% [5]. Diagnosis typically involves a combination of clinical evaluation, imaging methods (e.g., transvaginal ultrasound or CT scans), tumor marker assessment (e.g., CA-125), and histological confirmation via biopsy [6,7].

Treatment strategies for ovarian cancer depend on the tumor stage and histology. Standard first-line therapy involves surgical tumor debulking to confirm the histology and determine the stage, followed by adjuvant chemotherapy [7]. The standard chemotherapy regimen for epithelial ovarian cancers includes a platinum compound (cisplatin or carboplatin) combined with a taxane such as paclitaxel, administered in dose-dependent cycles [4,8]. Germ cell tumors are typically treated with a combination of bleomycin, etoposide, and cisplatin, while stromal tumors rarely require chemotherapy [9].

Chemotherapy regimens, while essential for treating ovarian cancer, come with a broad range of adverse effects due to the cytotoxic nature of these drugs. In addition to their desired effects on cancerous cells, chemotherapy also impacts rapidly dividing healthy cells, leading to a broad spectrum of side effects. These include neurotoxicity, hepatotoxicity, vomiting, nausea, and alopecia, among others [10]. These side effects arise from the drugs’ non-selective action on both cancerous and healthy cells that undergo rapid division, such as those in the hair follicles, gastrointestinal tract, and nervous system.

One of the most distressing side effects of cancer treatment is chemotherapy-induced alopecia (CIA), which profoundly affects patients’ quality of life, self-esteem, and body image [11,12]. CIA occurs due to the cytotoxic effects of chemotherapy on the anagen phase epithelial hair follicle stem cells, leading to anagen effluvium—a “process” in which the hair follicle bulb cells undergo apoptosis, resulting in hair breakage and shedding [13]. The overall incidence of CIA is estimated to be as high as 65% and varies according to the chemotherapy drug type and regimen [14].

Taxanes, such as paclitaxel and docetaxel, are among the most potent inducers of CIA due to their antimicrotubular action, which disrupts rapidly proliferating hair follicle matrix cells and damages stem cell-rich niches in the outer root sheath [14,15]. Platinum-based agents, such as carboplatin, often used in combination with taxanes, are also associated with CIA but are considered secondary contributors [16,17]. In general, a combination of drugs often causes hair loss that is more frequent and severe than the use of a single drug alone [18]. Additionally, the extent of hair loss has been reported to be influenced by factors such as dosage, administration regimen, age, comorbidities, and nutritional and hormonal status [19]. However, no detailed studies specifically exploring these factors in patients with ovarian cancer have been conducted.

Given the widespread use of chemotherapy for ovarian cancer and the significant burden of CIA on patients, understanding the incidence and severity of this side effect is critical. This study thoroughly reviews the available literature to provide insights into the incidence and severity of CIA in ovarian cancer patients, focusing on the effects of commonly used chemotherapeutic agents.

## 2. Methods

This scoping review aimed to map the available literature on CIA in ovarian cancer patients and to provide an overview of the incidence, severity, and proposed mechanisms across different chemotherapy regimens. This review was guided by the framework proposed by Arksey and O’Malley for scoping reviews, which involves identifying relevant studies, selecting eligible studies, charting the data, and summarizing the findings.

Searches were performed on PubMed and EMBASE databases using combinations of the following keywords: “alopecia”, “chemotherapy-induced alopecia”, “ovarian cancer”, and specific chemotherapy classes, including “platinum compounds” and “taxanes,” with Boolean operators (AND, OR). Inclusion criteria were limited to peer-reviewed clinical trials, cohort studies, and retrospective analyses that focused on treatment efficacy in ovarian cancer cohorts. Since there are limited studies exclusively investigating CIA in ovarian cancer patients, most studies analyzed provided quantitative data on the incidence and severity of CIA as a secondary outcome. Exclusion criteria included reviews, case reports, case series, commentaries, animal studies, and studies that focused solely on chemotherapy treatment without reporting CIA incidence.

Given the exploratory nature of this scoping review, an exact count of all initially identified studies is not available. Titles and abstracts were screened for relevance, and 27 full-text articles were reviewed. Of these, 18 studies met the inclusion criteria and were included in the final analysis. Key information was extracted from each study, including study characteristics, chemotherapy regimens and mechanism of action (MOA), and alopecia outcomes (incidence and severity).

The findings were summarized and are presented in Table 1, which provides an overview of chemotherapy regimens and associated alopecia outcomes. A comprehensive table delineating the selected articles and key extracted data is presented in Appendix A to enhance the transparency and reliability of the results.

## 3. Results

### 3.1. Platinum-Based Compounds: Cisplatin and Carboplatin

#### 3.1.1. MOA

Platinum-based (PB) agents such as cisplatin and carboplatin exert their cytotoxic effects by forming covalent cross-links within DNA strands, thereby preventing the separation of DNA during replication and transcription [20]. This disruption inhibits cell division, induces significant DNA damage, and activates apoptotic pathways. By inhibiting DNA repair mechanisms, these agents specifically target rapidly dividing cancer cells, making them highly effective for treating ovarian cancer.

#### 3.1.2. Regimen

Cisplatin and carboplatin are the most widely used PB compounds for this indication. Unlike fixed-dose chemotherapy agents, PB drugs require individualized dosing. The dose of cisplatin is calculated based on body surface area (BSA) and typically ranges from 10 to 100 mg/m^2^ [10]. For invasive epithelial ovarian cancer, the standard regimen is 75 mg/m^2^ on day 1, repeated every three weeks [21]. In contrast, carboplatin dosages are determined using the Calvert formula, which calculates the total dose in milligrams based on the drug dose, known as the area under the curve (AUC) and glomerular filtration rate (GFR), such that the total dose (mg) = target AUC × (GFR + 25). The target AUC typically ranges from 2 to 8 mg/mL·min per cycle, with regimens administered every three weeks [10].

#### 3.1.3. Alopecia

**Figure 1:**
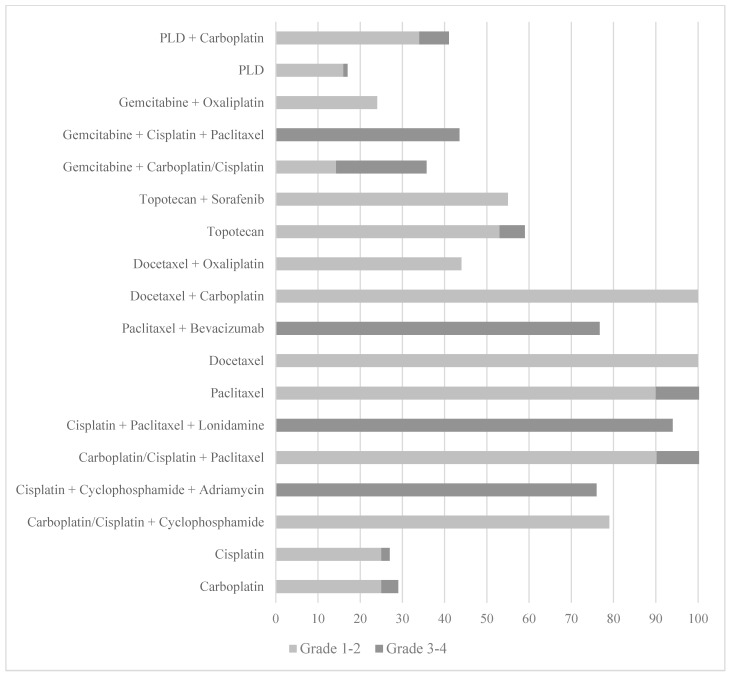
Summary of the incidence and severity of CIA in ovarian cancer patients across different chemotherapeutic regimens. The chart displays the percentage of patients experiencing grade 1–2 (mild to moderate) and grade 3–4 (severe) alopecia for both monotherapy and combination therapies.

While PB compounds are generally associated with lower rates of severe alopecia than other chemotherapy agents, they can still contribute to mild to moderate hair loss (Figure 1). Palmer et al. compared cisplatin monotherapy with a combination of cyclophosphamide and a platinum compound (either cisplatin or carboplatin) in advanced-stage newly diagnosed ovarian cancer patients [22]. The first group was treated with cisplatin (60 mg/m^2^) every two weeks for eight cycles, while the second group was treated with either cisplatin (75 mg/m^2^) or carboplatin (300 mg/m^2^) plus cyclophosphamide (600 mg/m^2^) every four weeks for six cycles. Alopecia (grade not specified) was only observed in 2% of patients in the cisplatin monotherapy group, while 79% developed alopecia in the combination PB and cyclophosphamide treatment regimen (Table 1).

The ICON3 study, a multicenter clinical trial, aimed to compare the safety and efficacy of paclitaxel combined with a PB compound with those of a non-taxane-containing regimen [23]. The treatments administered were as follows: carboplatin monotherapy (AUC 5) or cyclophosphamide, adriamycin, and cisplatin (CAP; 500 mg/m^2^, adriamycin 50 mg/m^2^, 50 mg/m^2^, respectively) combination vs. paclitaxel (175 mg/m^2^ given over a three-hour infusion) combined with carboplatin (AUC 5). The study demonstrated that carboplatin monotherapy caused significantly less grade 3–4 alopecia (4%) than combinations such as carboplatin plus paclitaxel (73–80%) or CAP (76%) (Table 1).

**Table 1:** Summary of the severity of chemotherapy-induced alopecia (CIA) by drug class in ovarian cancer patients.

Drug Class	Therapy Type	Key Agent	CIA Incidence	Alopecia Severity	References	Mechanism of CIA
Platinum Compounds	Monotherapy	Carboplatin	2.3–25%	Grade 2–4	[23,24,25]	DNA cross-linking, S-phase targeting
		Cisplatin	2–25%	Grade 1–4	[22,24]	
	Combination	Carboplatin/Cisplatin + Cyclophosphamide	18.4–79%	Grade 1–4	[22,26]	
		Cisplatin + Cyclophosphamide + Adriamycin	76%	Grade 3–4	[23]	
		Carboplatin/Cisplatin + Paclitaxel	34.2–90%	Grade 1–4	[23,24,26,27,28,29]	
		Cisplatin + Paclitaxel + Lonidamine	94%	Grade 3	[30]	
Taxanes	Monotherapy	Paclitaxel	62.8–79%	Grade 3	[31,32]	Microtubule stabilization, mitotic arrest
		Docetaxel	100%	Grade 1–2	[33]	
	Combination	Paclitaxel + Bevacizumab	76.7%	Grade 3	[32]	
		Docetaxel + Carboplatin	100%	Grade 1–2	[34]	
		Docetaxel + Oxaliplatin	44.2%	Grade 1–2	[35]	
Topoisomerase Inhibitors	Monotherapy	Topotecan	49–53%	Grade 1–4	[36,37]	DNA damage due to enzyme inhibition
	Combination	Topotecan + Sorafenib	55%	Grade 1–2	[37]	
Antimetabolites	Combination	Gemcitabine + Carboplatin/Cisplatin	14.3–21.4%	Grade 2–3	[25,38]	DNA synthesis inhibition and chain termination due to enzyme inhibition
		Gemcitabine + Cisplatin + Paclitaxel	43.5%	Grade 3	[39]	
		Gemcitabine + Oxaliplatin	24%	Grade 2	[40]	
Anthracyclines	Monotherapy	PLD	16%	Grade 1–4	[36]	Targeted delivery reduces systemic toxicity
	Combination	PLD + Carboplatin	7–34%	Grade 1–4	[28,29,41]	

Another study evaluated cisplatin combination regimens, comparing cisplatin and cyclophosphamide with cisplatin and paclitaxel for stages III and IV ovarian cancer treatment [26]. The first group received intravenous cisplatin (75 mg/m^2^) and cyclophosphamide (750 mg/m^2^) every three weeks for a total of six cycles. The second group was administered intravenous cisplatin (75 mg/m^2^) and paclitaxel (135 mg/m^2^ over 24 h) every three weeks for six cycles. In the cisplatin and cyclophosphamide combination group, 13.4% of women developed grade 2 alopecia, while 5% had grade 1 alopecia. Conversely, patients in the cisplatin and paclitaxel combination group had a higher incidence of moderate alopecia, with 29.3% reporting grade 2 alopecia and 4.9% grade 1 alopecia (Table 1). Historically, agents such as cyclophosphamide and chlorambucil have been used as first-line treatments for ovarian cancer but are currently obsolete in the management of ovarian cancer [42].

The Multicenter Italian Trial in Ovarian Cancer (MITO-7) trial further explored the incidence of alopecia at different dosing schedules [27]. Patients were randomized to receive carboplatin (AUC 6) plus paclitaxel (175 mg/m^2^ over three hours) every three weeks for six cycles or a weekly regimen comprising carboplatin (AUC 2) plus paclitaxel (60 mg/m^2^ over one hour) for 18 weeks. Although progression-free survival was similar between regimens, the weekly schedule resulted in significantly less grade 2 alopecia (29%) than the every three-week regimen (59%). However, mild alopecia (grade 1) was reported more frequently in the weekly regimen group (21%) than in the three-week regimen group (7%). This suggests that treatment frequency plays a crucial role in the severity of alopecia, with a three-week schedule leading to more severe hair loss.

Another combination regimen employed to treat advanced ovarian cancers is cisplatin, paclitaxel, and lonidamine [30]. Patients were administered paclitaxel intravenously (135 mg/m^2^ over three hours) on day one, followed by intravenous cisplatin (75 mg/m^2^) on day two, and lonidamine orally (450 mg/daily) for six consecutive days (starting two days prior to chemotherapy), every three weeks for six cycles. Grade 3 alopecia was reported in 94% of patients (Table 1), demonstrating that although progression-free survival improved with the addition of lonidamine, increased severity and incidence of alopecia were equally observed in patients.

### 3.2. Taxanes: Paclitaxel and Docetaxel

#### 3.2.1. MOA

Paclitaxel and docetaxel exert their effects by binding to the β-tubulin subunit of microtubules, stabilizing them, and preventing their disassembly [43]. This stabilization disrupts the normal dynamics of microtubule assembly and disassembly, which are essential for cell division. By interfering with mitosis, taxanes cause cell cycle arrest, ultimately leading to apoptosis [44].

#### 3.2.2. Regimen

Paclitaxel emerged in the early 1990s as a pivotal chemotherapeutic agent for ovarian cancer, first gaining prominence in the ICON3 trial. It is commonly used in combination with cisplatin or carboplatin following initial surgery and serves as a mainstay in the treatment of recurrent or refractory ovarian cancer, either as monotherapy or in combination with other agents [44].

Traditionally, it has been administered at 175 mg/m^2^ every three weeks, particularly when combined with PB agents. Recently, paclitaxel has undergone significant regimen optimization to balance efficacy and toxicity, with a current administration of 80 mg/m^2^ weekly. This weekly schedule with a reduced dosage has become the preferred approach because of its comparable efficacy and more favorable toxicity profile, including reduced neuropathy and other adverse effects [45].

As a single agent for advanced ovarian cancer, docetaxel is administered at 100 mg/m^2^ every three weeks [46]. More recently, docetaxel has been added to combination regimens with platinum compounds such as carboplatin. The typical dosage regimen for stages IIB to IV epithelial ovarian cancers treated with this combination is intravenous docetaxel (30 mg/m^2^) administered weekly and carboplatin (AUC 5) given on day 1 every three weeks for at least six cycles [47].

#### 3.2.3. Alopecia

Alopecia is one of the most distressing side effects of taxane-based therapy. Paclitaxel is associated with high rates of complete but reversible alopecia (Figure 1), particularly with prior treatment or in combination with platinum-based agents [31].

The ICON4/AGO-OVAR-2.2 trial, a multicenter randomized trial, compared paclitaxel combined with PB compounds to conventional PB monotherapy in women with platinum-sensitive relapsed ovarian cancer [24]. Patients were assigned to a minimum of six cycles, PB monotherapy with either carboplatin (AUC 5) or cisplatin (75 mg/m^2^), or paclitaxel administered at a dosage of 175 mg/m^2^ given over a three-hour infusion combined with carboplatin (AUC 5) or cisplatin (50 mg/m^2^). The findings demonstrated that the addition of paclitaxel to PB chemotherapy significantly increased the incidence of grade 2–4 alopecia (86%) compared with PB monotherapy regimens (25%) (Table 1). Despite the higher incidence of alopecia, combination therapy improved survival outcomes, making the trade-off in side effects acceptable for many patients.

To evaluate the effects of dosage frequency with paclitaxel, a randomized multicenter study by Rosenberg et al. investigated paclitaxel-naïve women with recurrent or progressive ovarian cancer who had previously received PB chemotherapy [31]. The patients were randomized to receive paclitaxel either weekly (67 mg/m^2^ over three hours) or every three weeks (200 mg/m^2^ over three hours). The study found that grade 3 alopecia occurred more frequently with the three-week regimen (79%) than with weekly treatment (46%) (Table 1). Additionally, cumulative grade 1–3 alopecia showed comparable incidence rates between the every three-weeks dosing schedule and the weekly regimen, at 90% and 82%, respectively. These findings suggest that while the overall incidence of alopecia is similar between dosing regimens, a less frequent but higher-dose paclitaxel regimen is associated with a greater severity of alopecia.

An alternative formulation of paclitaxel has also been examined. Albumin-bound paclitaxel (ABP) was tested in combination with bevacizumab, a monoclonal antibody, for the treatment of platinum-resistant recurrent ovarian cancer [32]. ABP is an innovative formulation of paclitaxel that uses albumin as a carrier. This new preparation demonstrated enhanced tumor-fighting capabilities and reduced side effects compared to conventional paclitaxel formulations [48,49]. ABP was intravenously administered (135–175 mg/m^2^) over 30 min once a day, and bevacizumab was infused for 90 min (7.5 mg/kg) every three weeks for six courses. Grade 3 alopecia was reported in 76.7% of the subjects in the combination group compared with 62.8% in the ABP monotherapy group (Table 1).

A phase II trial of patients with platinum-refractory advanced ovarian cancer examined the efficacy and toxicity of docetaxel treatment [33]. Docetaxel was administered at a dose of 100 mg/m^2^ intravenously over one hour every three weeks. All patients developed alopecia, with grade 2 alopecia observed in 92% of subjects and grade 1 in 8% (Table 1).

In a clinical trial by Aoki et al., docetaxel was administered in combination with carboplatin in chemotherapy-naïve patients with stage IC to IV ovarian cancer [34]. Docetaxel (70 mg/m^2^) and carboplatin (AUC 5) were administered consecutively on day one of a three-week cycle for five cycles. Grade 2 alopecia was observed in 77% of the subjects, and grade 1 alopecia was observed in 23% (Table 1).

Another combination regimen was examined by Ferrandina et al. in a phase II study that included patients with platinum-sensitive recurrent ovarian cancer [35]. Intravenous administration of docetaxel (75 mg/m^2^) over one hour, followed by oxaliplatin (100 mg/m^2^) over two hours, was given on day one of a three-week cycle. This combination regimen resulted in grade 2 alopecia in 34.9% of the patients and grade 1 alopecia in 9.3% (Table 1). These findings suggest that this combination has a favorable non-hematologic toxicity profile, with lower rates of hair loss than PB–docetaxel regimens.

### 3.3. Topoisomerase I Inhibitors: Topotecan

#### 3.3.1. MOA

Topotecan is a well-studied treatment for ovarian cancer, particularly in cases resistant to PB therapies. It functions similarly to paclitaxel and does not develop cross-resistance to paclitaxel or platinum [50].

Topotecan inhibits topoisomerase I, an enzyme essential for relieving torsional strain, thereby facilitating DNA replication [51]. By stabilizing the enzyme–DNA complex, topotecan prevents the re-ligation of single-strand breaks in DNA. As the replication fork encounters these stabilized complexes, double-strand breaks occur, leading to the accumulation of DNA damage, which triggers apoptosis. This mechanism specifically targets rapidly dividing cells, thereby enhancing the efficacy of topotecan against ovarian cancer.

#### 3.3.2. Regimen

The standard dosing regimen for treating relapsed ovarian cancer is 1.5 mg/m^2^ daily for five consecutive days in a three-week cycle. However, a lower dose of 1.25 mg/m^2^ daily is commonly used in clinical settings to mitigate side effects while maintaining therapeutic efficacy [52].

#### 3.3.3. Alopecia

Alopecia is a notable adverse effect of topotecan, though less severe compared to taxanes (Figure 1). In a phase III randomized clinical trial, Gordon et al. compared topotecan with pegylated liposomal doxorubicin (PLD) in patients with recurrent epithelial ovarian carcinoma [36]. Patients received either topotecan (1.5 mg/m^2^) daily for five consecutive days every three weeks or PLD (50 mg/m^2^) over a one-hour infusion every four weeks. The study reported significantly higher rates of alopecia in patients treated with topotecan (49%) than in those treated with PLD (16%) (Table 1). Severe alopecia (grade 3–4) was observed in 6% of the subjects treated with topotecan and only in 1% of the PLD-treated individuals (Figure 1).

A phase II, multicenter, randomized, double-blind, placebo-controlled trial investigated the treatment of platinum-resistant ovarian cancers with a combination of topotecan and sorafenib, a multikinase inhibitor [37]. Patients were administered topotecan (1.25 mg/m^2^) intravenously over 30 min on days 1–5 followed either by placebo or oral sorafenib (400 mg) twice daily on days 6–15, every three weeks for up to six cycles. Grade 1–2 alopecia was reported in 55% of the patients in the combination group and 53% of the patients in the topotecan monotherapy group (Table 1). These findings suggest that topotecan, rather than sorafenib, is the primary contributor to non-hematological toxicity, specifically, alopecia.

### 3.4. Antimetabolites: Gemcitabine

#### 3.4.1. MOA

Gemcitabine is a nucleoside analog that interferes with DNA synthesis and repair. Once inside the cells, it undergoes phosphorylation to its active metabolites gemcitabine diphosphate (dFdCDP) and gemcitabine triphosphate (dFdCTP). dFdCDP inhibits ribonucleotide reductase, thereby reducing the production of the deoxynucleotides required for DNA synthesis. Conversely, dFdCTP is incorporated into growing DNA strands during replication, causing chain termination. Together, these mechanisms lead to the accumulation of DNA damage and trigger cell death, effectively targeting ovarian cancer cells [53].

#### 3.4.2. Regimen

Gemcitabine is commonly used in platinum-sensitive recurrent ovarian cancer and is typically administered in a three-week cycle at a dose of 1000 mg/m^2^ on days one and eight [25]. Gemcitabine is often combined with carboplatin (AUC 4) on day one, every three weeks for up to six cycles, to enhance its efficacy in platinum-sensitive and refractory cases [54]. Less commonly, a four-week cycle with dosing on days 1, 8, and 15 is employed in certain clinical trials or for specific patient needs [55].

#### 3.4.3. Alopecia

Alopecia associated with gemcitabine is less common than the previously discussed chemotherapeutic regimens (Figure 1). A phase II study examined the efficacy and toxicity of gemcitabine combined with cisplatin as first-line treatment for chemotherapy-naïve patients with stage III or IV epithelial ovarian cancer [38]. Patients received cisplatin (75 mg/m^2^) followed by gemcitabine (1250 mg/m^2^) on days one and eight of a three-week cycle. This combination resulted in 21.4% of the participants developing grade 3 alopecia (Table 1).

In a phase III randomized trial conducted by Pfisterer et al., patients with platinum-sensitive recurrent ovarian cancer were treated with either gemcitabine (1000 mg/m^2^ on days one and eight) and carboplatin (AUC 4 on day one) or carboplatin monotherapy (AUC 5 on day one) [25]. Grade 2 alopecia occurred in 14.3% of the subjects in the combination group compared to 2.3% of those receiving carboplatin monotherapy (Table 1). The study demonstrated that the combination of gemcitabine and carboplatin had a superior response rate for treating ovarian cancer compared to carboplatin monotherapy, with acceptable toxicity, highlighting the relatively mild cytotoxic effect of gemcitabine on hair follicle cells.

Gemcitabine has also been combined with oxaliplatin for taxane- and platinum-resistant ovarian cancers. A phase II study tested gemcitabine (1000 mg/m^2^ over 100 min) administered on days one and eight in addition to oxaliplatin (100 mg/m^2^) on day one [40]. Grade 2 alopecia was observed in 24% of the patients (Table 1), although most patients initiated the study with pre-existing alopecia due to previous treatments.

A triple combination of gemcitabine, paclitaxel, and cisplatin was also evaluated in a multicenter, open-label, phase II trial of chemotherapy-naïve patients with advanced ovarian cancer [39]. Patients were administered cisplatin (70 mg/m^2^) on day one, followed by paclitaxel (80 mg/m^2^) and gemcitabine (1000 mg/m^2^) on days one and eight, every three weeks. Grade 3 alopecia was reported in 43.5% of the patients (Table 1), with no grade 4 alopecia observed.

### 3.5. Anthracyclines: Pegylated Liposomal Doxorubicin

#### 3.5.1. MOA

Doxorubicin exerts its anticancer effects by intercalating into adjacent base pairs of DNA, disrupting replication and transcription, and inhibiting topoisomerase II, resulting in double-strand breaks and apoptosis [56]. While both doxorubicin and its pegylated liposomal formulation (PLD) share this mechanism, PLD differs in its formulation and toxicity profile. Encapsulation of doxorubicin in liposomes coated with polyethylene glycol enhanced tumor-specific delivery, prolonged circulation time, and reduced off-target effects, particularly cardiotoxicity and alopecia [57].

#### 3.5.2. Regimen

The standard dosing of PLD ranges between 30 and 50 mg/m^2^ every four weeks, with studies showing that its concentration in tumors can be 10-fold higher than that of free doxorubicin [28,56]. This enhanced tumor-targeting formulation reduces systemic toxicity while maintaining therapeutic efficacy.

#### 3.5.3. Alopecia

The incidence of alopecia is notably lower with PLD than with the traditional regimens (Figure 1). A meta-analysis by Li et al. found that PLD combined with carboplatin significantly decreased the risk of alopecia (RR 0.09; 95% CI 0.07–0.12; *p* < 0.01) [41]. A systematic review by Lawrie et al. compared the combination of PLD and carboplatin with paclitaxel and carboplatin for relapsed epithelial ovarian cancer [29]. They reported that grade 2 alopecia was observed in 7.6% of the patients undergoing treatment with the PLD and carboplatin combination, compared to 84% for those receiving the paclitaxel and carboplatin combination (Table 1).

A study by Pujade-Lauraine et al. examined patients with platinum-sensitive ovarian cancers in late relapse, treated with the PLD regimen, carboplatin (AUC 5) plus PLD (30 mg/m^2^) administered every four weeks, or the carboplatin (AUC 5) plus paclitaxel (175 mg/m^2^) regimen every three weeks, for a minimum of six cycles [28]. Alopecia was reported in 90.2% of the patients undergoing carboplatin and paclitaxel combination treatment, whereas only 34% of the patients in the PLD and carboplatin combination group developed alopecia (Table 1). More significantly, higher rates of moderate-to-severe alopecia were observed in the carboplatin and paclitaxel combination group (83.6%) than in the PLD combination regimen (7%). These findings highlight that the combination of PLD and carboplatin is associated with a markedly lower incidence of moderate-to-severe alopecia and presents a milder alopecia side effect profile.

## 4. Discussion

Chemotherapy regimens used for ovarian cancer can cause a range of adverse effects across multiple organs, as part of a broad toxicity profile [20]. Among these, CIA stands out not only because of its visibility but also due to its emotional and psychological toll [18,57,58]. The relationship between chemotherapy regimens used for ovarian cancer and their influence on CIA is complex and is influenced by differences in mechanisms of action, dosing schedules, and prior treatment history. Prior chemotherapy can exacerbate alopecia in subsequent regimens [36]. Patients with previous exposure to platinum-based agents or taxanes may experience more severe hair loss due to pre-existing follicular damage, making them more vulnerable to the cytotoxic effects of new treatments. CIA significantly diminishes the quality of life of patients, making it a key consideration throughout treatment. 

Taxanes pose the highest risk for CIA, with the majority of patients treated with paclitaxel or docetaxel experiencing severe alopecia (Figure 1). Their upstream mechanism of disrupting microtubule assembly broadly affects both cancer cells and hair follicles. In contrast, platinum-based compounds, such as carboplatin and cisplatin, induce significantly less and milder hair loss, as their DNA cross-linking mechanism during the S-phase of the cell cycle targets rapidly dividing cancerous cells more selectively [23,59].

Similarly, topoisomerase I inhibitors (topotecan), antimetabolites (gemcitabine), and anthracyclines (pegylated liposomal doxorubicin) are associated with milder CIA than taxanes, owing to their greater specificity in targeting cancer cells. Topoisomerase I inhibitors leverage the overexpression of topoisomerase I in ovarian tumors, resulting in fewer side effects including alopecia [60,61]. Gemcitabine, another S-phase-specific agent, has a low incidence of alopecia and a favorable side effect profile, making it a particularly appealing option for patients prioritizing a reduction in hair loss [53]. PLD, with targeted delivery, has a notably low incidence of alopecia [56]. In a phase III trial of taxane-refractory breast cancer, only 3% of PLD-treated patients experienced alopecia [62]. Its liposomal formulation reduces systemic toxicity and hair follicle damage, as evidenced by a study in advanced soft tissue sarcoma, where 6% of PLD-treated patients reported alopecia compared to 86% with conventional doxorubicin [63].

Cumulative toxicity from combination chemotherapy regimens often worsens CIA by compounding the damage to rapidly dividing cells such as hair follicles (Table 2).

Different chemotherapeutic agents target distinct cellular processes, which, when utilized in combination, can exert a more significant and comprehensive effect on the hair follicle. For instance, the addition of paclitaxel to platinum-based chemotherapy increased alopecia rates from 25% to 86% in the ICON4/AGO-OVAR-2.2 trial [24]. Similarly, the ICON3 trial demonstrated higher rates of alopecia with paclitaxel–carboplatin combinations (73%) than with carboplatin monotherapy (4%) [23]. This effect is further magnified in triple combination therapies. For example, the combination of cisplatin, paclitaxel, and lonidamine resulted in 94% of patients developing grade 3 alopecia [30]. Paclitaxel disrupts microtubule formation, which is essential for cell division, leading to damage in rapidly dividing cells like those in hair follicles. Platinum agents like carboplatin cause DNA cross-linking, which inhibits DNA replication and results in cellular damage, while lonidamine inhibits mitochondrial-bound hexokinase, enhancing the damage to proliferative tissues. When used together, these agents exacerbate the overall toxicity by targeting different, yet complementary, mechanisms, resulting in compounded damage to hair follicle cells. However, certain combinations, such as PLD with carboplatin, had significantly lower risks of alopecia, as observed by Li et al. [41]. This is likely due to PLD’s targeted delivery, which reduces systemic toxicity and spares hair follicles. These findings suggest that while taxane-based combinations tend to exacerbate alopecia, regimens such as PLD with platinum may offer a more tolerable alternative.

The severity of CIA also varies with treatment frequency. High-dose regimens administered every three weeks caused greater toxicity to hair follicle cells, particularly during the anagen phase, resulting in more severe hair loss (Table 2). Conversely, weekly regimens with lower fractionated doses allow for partial follicular recovery and reduce the cumulative damage. For instance, in the MITO-7 trial, a weekly carboplatin–paclitaxel regimen caused significantly less alopecia (29%) than the standard every-three-week schedule (59%) [27]. Similarly, Rosenberg et al. found that grade 3 alopecia was more frequent in patients receiving paclitaxel every three weeks (79%) than in those receiving weekly paclitaxel (46%) [31]. These findings highlight the potential of weekly regimens to reduce alopecia severity, although the evidence for other chemotherapy classes is limited.

Treatment-related side effects, such as CIA, can profoundly affect patients’ self-esteem, emotional well-being, and social interactions [18,57,58]. Clinicians should provide thorough counseling to explain the potential timing, extent, and permanence of hair loss. Offering support through interventions such as scalp cooling devices, which reduce follicular damage, and hair regrowth treatments can help mitigate some of the distress caused by hair loss [57].

Our findings may be limited by the lack of studies specifically focusing on CIA in ovarian cancer, as most data come from studies where alopecia is a secondary outcome. Variations in reporting standards and grading scales further complicate comparisons. Future research should prioritize evaluating CIA in ovarian cancer using standardized methods of assessment, along with mechanistic studies exploring how chemotherapy regimens, such as dosing frequency, can be adjusted to minimize alopecia while maintaining efficacy.

## 5. Conclusions

Chemotherapy-induced alopecia (CIA) is a significant adverse effect of ovarian cancer treatments, profoundly impacting patients’ quality of life. This review highlights the varying incidence and severity of CIA across commonly used chemotherapy regimens, with taxanes and platinum-based combinations presenting the highest risk. Factors such as dosing schedules, drug combinations, and prior treatment exposure further influence the extent of hair loss. Strategies such as weekly dosing schedules and targeted therapies, like pegylated liposomal doxorubicin, show promise in reducing CIA without compromising therapeutic outcomes. Addressing CIA is essential not only for improving patient well-being but also for enhancing adherence to treatment. Future research should prioritize standardized assessment methods and the development of interventions to mitigate CIA while maintaining efficacy in ovarian cancer care.

## Figures and Tables

**Table 2:** Overview of key factors influencing the severity of chemotherapy-induced alopecia (CIA) in ovarian cancer patients.

Factor	Impact on Alopecia	Evidence
Drug Combination	Combination therapy of taxanes and platinum-based compounds increase the incidence of alopecia.	Paclitaxel: 25% (monotherapy) vs. 86% (combination with PB compound) [24]. Carboplatin: 4% (monotherapy) vs. 73% (combination with paclitaxel) [23].
Dose and Dosing Schedule	Higher doses administered less frequently result in more severe alopecia.	Carboplatin–paclitaxel: 29% (weekly) vs. 59% (every three weeks) [27]. Paclitaxel: 46% (weekly) vs. 79% (every three weeks) [31].
Prior Chemotherapy	Prior chemotherapy exacerbates the incidence and severity of alopecia.	Alopecia incidence: 25% (paclitaxel with chemotherapy-naïve patients) vs. 46–79% (paclitaxel with prior PB exposure) [24,31].
Drug Delivery Methods	Targeted therapy (e.g., liposomal encapsulation) reduces the incidence of alopecia.	PLD leads to lower alopecia rates (6%) compared to conventional doxorubicin (86%) in soft tissue sarcoma patients [63]. Alopecia incidence: PLD + carboplatin (7.6–34%) vs. paclitaxel + carboplatin (84–90.2%) [28,29].

## Appendix A

**Table A1:** Summary of all studies analyzed in this review.

		Author	Sample Size	Treatment Regimen		Primary Endpoint	Secondary Endpoint	Alopecia Incidence and Severity
				Monotherapy	Combination Therapies			
**Platinum compounds**	Cisplatin	Palmer et al. [22]	70	Cisplatin (60 mg/m^2^) q2W for eight cycles	Cisplatin (75 mg/m^2^) or carboplatin (300 mg/m^2^) + cyclophosphamide (600 mg/m^2^) q4W for six cycles	OS	Toxicity	Alopecia (grade not specified) observed in 2% of patients in the cisplatin monotherapy group, while 79% developed alopecia in the combination group.
		McGuire et al. [26]	386	N/A	Cisplatin (75 mg/m^2^) + cyclophosphamide (750 mg/m^2^) q3W for six cycles	OS	PFS, CRR, pRR, toxicity, feasibility of treatment completion	In the cisplatin and cyclophosphamide combination group, 13.4% of women developed grade 2 alopecia, while 5% had grade 1 alopecia. Conversely, patients in the cisplatin and paclitaxel combination group had a higher incidence of moderate alopecia, with 29.3% reporting grade 2 alopecia and 4.9% grade 1 alopecia.
					Cisplatin (75 mg/m^2^) + paclitaxel (135 mg/m^2^) q3W for six cycles			
		De Lena et al. [30]	35	N/A	Paclitaxel (135 mg/m^2^) on day 1 + cisplatin (75 mg/m^2^) on day 2 + lonidamine (450 mg/daily for 6 days), q3W for six cycles	ORR as defined by CR and PR, PFS	OS, toxicity	Grade 3 alopecia was reported in 94% of patients.
	Carboplatin	International Collaborative Ovarian Neoplasm Group [23]	2074	Carboplatin (AUC 5), q3W for six cycles	CAP: Cyclophosphamide (500 mg/m^2^) + adriamycin (50 mg/m^2^) + cisplatin (50 mg/m^2^), q3W for six cycles	OS	PFS, toxicity	Carboplatin monotherapy caused significantly less grade 3–4 alopecia (4%) than CAP (76%) and CP (73–80%).
					CP: Carboplatin (AUC 5) + paclitaxel (175 mg/m^2^), q3W for six cycles			
		Pignata et al. [27]	810	N/A	Carboplatin (AUC 6) + paclitaxel (175 mg/m^2^) q3W for six cycles	PFS and QoL	OS, ORR, toxicity, treatment feasibility	Weekly schedule resulted in significantly less grade 2 alopecia (29%) than every three-week regimen (59%). However, mild alopecia (grade 1) was reported more frequently in the weekly regimen group (21%) than in the three-week regimen group (7%).
					Carboplatin (AUC 2) + paclitaxel (60 mg/m^2^) q1W for 18 weeks			
**Taxanes**	Paclitaxel	Parmar et al. [24]	802	Carboplatin (AUC 5), q3W for 3–8 cycles	Carboplatin (AUC 5) + paclitaxel (175–185 mg/m^2^) q3W for 3–8 cycles	OS	PFS, QoL, toxicity	The addition of paclitaxel to PB chemotherapy significantly increased the incidence of grade 2–4 alopecia (86%) compared with PB monotherapy regimens (25%).
				Cisplatin (75 mg/m^2^) q3W for 3–8 cycles	Cisplatin (50 mg/m^2^) + paclitaxel (175–185 mg/m^2^) q3W for 3–8 cycles			
		Rosenberg et al. [31]	208	Paclitaxel (67 mg/m^2^) q1W (range of 1–16 cycles; median of 5.7)	N/A	ORR as defined by CR and PR	Response duration, TtP, OS, toxicity and safety	Grade 3 alopecia occurred more frequently with the three-week regimen (79%) than with weekly treatment (46%). Cumulative grade 1–3 alopecia showed comparable incidence rates between the every three-weeks dosing schedule and the weekly regimen, at 90% and 82%, respectively.
				Paclitaxel (200 mg/m^2^) q3W (range of 1–17 cycles; median of 7)				
		Liu et al. [32]	86	Albumin-bound paclitaxel (ABP) (135–175 mg/m^2^) q3W for six cycles	ABP (135–175 mg/m^2^) + bevacizumab (7.5 mg/kg) q3W for six cycles	ORR as defined by CR and PR	DCR, PFS, OS, changes in serum CA125 levels, toxicity and safety	Grade 3 alopecia was reported in 76.7% of the subjects in the combination group compared with 62.8% in the ABP monotherapy group.
	Docetaxel	Francis et al. [33]	25	N/A	Docetaxel (100 mg/m^2^) q3W for 1–10 cycles (median of 4 cycles)	ORR as defined by CR and PR	Response duration, TtP, toxicity, median survival duration	All patients developed alopecia, with grade 2 alopecia observed in 92% of the subjects and grade 1 in 8%.
		Aoki et al. [34]	26	N/A	Docetaxel (70 mg/m^2^) + carboplatin (AUC 5) q3W for five cycles	PFS	OS, ORR, toxicity, feasibility and tolerability	Grade 2 alopecia was observed in 77% of the subjects and grade 1 alopecia observed in 23%.
		Ferrandina et al. [35]	43	N/A	Docetaxel (75 mg/m^2^) + oxaliplatin (100 mg/m^2^) q3W for six cycles	CRR as defined by CR and PR, and TtP	OS, toxicity and safety	This combination regimen resulted in grade 2 alopecia in 34.9% of the patients and grade 1 alopecia in 9.3%.
**Topoisomerase I inhibitors**	Topotecan	Gordon et al. [36]	481	Topotecan (1.5 mg/m^2^) daily for 5 days, q3W for eight cycles	N/A	PFS	OS, ORR as defined by CR or PR, toxicity and safety, health-related QoL	The study reported significantly higher rates of alopecia (all grades) in patients treated with topotecan (49%) than in those treated with PLD (16%). Severe alopecia (grade 3–4) was more common in the topotecan group (6%) than in the PLD-treated group (1%).
				Pegylated liposomal doxorubicin (PLD) (50 mg/m^2^), q4W for six cycles				
		Chekerov et al. [37]	174	N/A	Topotecan (1.25 mg/m^2^) daily for 5 days + placebo, q3W for six cycles	PFS	OS, ORR as defined by CR and PR, duration of response, TtP, health-related QoL, toxicity and safety, tolerability of treatment	Mild-to-moderate alopecia was reported in 55% of patients in the topotecan and sorafenib combination group and 53% of patients in the topotecan and placebo group.
					Topotecan (1.25 mg/m^2^) daily for 5 days + sorafenib (400 mg) BID on days 6–15, q3W for six cycles			
**Antimetabolites**	Gemcitabine	Belpomme et al. [38]	42	N/A	Cisplatin (75 mg/m^2^) on day 1 + gemcitabine (1250 mg/m^2^) on days 1 and 8, q3W for six cycles	ORR as defined by CR and PR	PFS, OS, duration of response, toxicity and safety	This combination resulted in 21.4% of the participants developing grade 3 alopecia.
		Pfisterer et al. [25]	356	Carboplatin (AUC 5) on day 1, q3W for six cycles	Carboplatin (AUC 4) on day 1 + gemcitabine (1000 mg/m^2^) on days 1 and 8, q3W for six cycles	PFS	OS, ORR as defined by CR and PR, duration of response, toxicity and safety, QoL	Grade 2 alopecia occurred in 14.3% of the subjects in the combination group compared to 2.3% in those receiving carboplatin monotherapy. Grade 1 alopecia was reported in 34.9% in the experimental arm and 15.5% in the control arm.
		Ray-Coquard et al. [40]	50	N/A	Gemcitabine (1000 mg/m^2^) on days 1 and 8 + oxaliplatin (100 mg/m^2^) on day 1, q3W for six cycles	ORR as defined by CR and PR	TtP, PFS, duration of response, OS, toxicity and safety	Grade 2 alopecia was observed in 24% of the patients, although most patients initiated the study with pre-existing alopecia due to previous treatments.
		Gupta et al. [39]	46	N/A	Cisplatin (70 mg/m^2^) on day 1 + paclitaxel (80 mg/m^2^) + gemcitabine (1000 mg/m^2^) on days 1 and 8, q3W for six cycles	ORR as defined by CR and PR	PFS, TtPD, OS, duration of response, toxicity and safety, pCR, treatment feasibility	Grade 3 alopecia was reported in 43.5% of the patients, with no grade 4 alopecia observed.
**Anthracyclines**	Pegylated liposomal doxorubicin	Pujade-Lauraine et al. [28]	976	N/A	Carboplatin (AUC 5) + PLD (30 mg/m^2^), q4W for six cycles	PFS	OS, QoL, toxicity and safety, disease progression	Alopecia (all grades) was reported in 90.2% of patients undergoing carboplatin and paclitaxel combination treatment, whereas only 34% of the patients in the PLD and carboplatin combination group developed alopecia. Higher rates of moderate-to-severe alopecia were observed in the carboplatin and paclitaxel combination group (83.6%) than in the PLD combination regimen (7%).
					Carboplatin (AUC 5) + paclitaxel (175 mg/m^2^), q3W for six cycles			

Clinical response rate (CRR); overall response rate (ORR); complete response (CR); partial response (PR); progression-free survival (PFS); time to progression or disease (TtPD); overall survival (OS); quality of life (QoL); pathological complete response (pCR); pathological response rate (pRR); disease control rate (DCR).
